# A “Diaploma”

**Published:** 1866-12

**Authors:** 


### A "Diaploma."

The following document, *literatim et punctuatim*, was found in the pocket of a "doctor" who recently died in one of our public institutions. It is a good specimen of the method of manufacturing some of the "doctors" who go abroad and practice on "American" diplomas." Such manufactories exist under one name or another in most of our large cities:

"New Orleans.

"Dr. William H. Rathbone.

"This diaploma I do give from my right hand and do solemnly swear that Dr. ——— ——— has served three years and one month in our disecting rooms we the undersigned do solemnly swear that ——— ——— is truly a brother of doing good—we know that he can reduce pragnacy or anything belonging to midwifery we have also tryed his Skill with old chronic diseases—very much to our Satisfaction as he has Been Successful in every case where the medicine has Been used according to his directions—as for medicine he is a self educated man he understands the human sistem thourily and is prpaed to give explinations to lecture apen the Subject—his remrcable and powerful Skill has Been tested here with us—we feel willing to recomment him above all others thus far graduates from our desecting Rooms—we think there is something in his powers aforeseen that we cannot account for the following is a true discription as we are able to give of him his complection is hard to get at his hair is of a dark chestnut color his beard of redish cast his eyes of brilliant colors the hair on his chest are black he has a mark on the upper lip and two of his nuckles on his right hand are knocked off his hith five feet seven in and half and weight about a hundred and fifty three pounds and if the above marks are not found on him and a true discription of him—we can not allow him this diaploma But if this discription will answer we allow him to be a faithful Brother to his profession and may god bless him in doing good in this great and glorius heres is our well wishes now and forever.

Prof William H Rathbone
Henry Rodwell M D
Edward Willard M. D."

